# Perspectives on reproductive healthcare delivered through a basic package of health services in Afghanistan: a qualitative study

**DOI:** 10.1186/1472-6963-14-359

**Published:** 2014-08-28

**Authors:** Natasha Howard, Aniek Woodward, Dhrusti Patel, Ahmad Shafi, Lisa Oddy, Annemarie ter Veen, Nooria Atta, Egbert Sondorp, Bayard Roberts

**Affiliations:** London School of Hygiene and Tropical Medicine, London, UK; King’s College London, London, UK; Duke University, Durham, North Carolina USA; Rumi Consultancy, Kabul, Afghanistan; McGill University, Montreal, Canada; Royal Tropical Institute, Amsterdam, the Netherlands; Kabul Medical University, Kabul, Afghanistan

**Keywords:** BPHS, SRH, Reproductive health, Health-system framework, Afghanistan

## Abstract

**Background:**

Contracting-out non-state providers to deliver a minimum package of essential health services is an increasingly common health service delivery mechanism in conflict-affected settings, where government capacity and resources are particularly constrained. Afghanistan, the longest-running example of Basic Package of Health Services (BPHS) contracting in a conflict-affected setting, enables study of how implementation of a national intervention influences access to prioritised health services. This study explores stakeholder perspectives of sexual and reproductive health (SRH) services delivered through the BPHS in Afghanistan, using Bamyan Province as a case study.

**Methods:**

Twenty-six in-depth interviews were conducted with health-system practitioners (e.g. policy/regulatory, middle management, frontline providers) and four focus groups with service-users. Inductive thematic coding used the WHO Health System Framework categories (i.e. service delivery, workforce, medicines, information, financing, stewardship), while allowing for emergent themes.

**Results:**

Improvements were noted by respondents in all health-system components discussed, with significant improvements identified in service coverage and workforce, particularly improved gender balance, numbers, training, and standardisation. Despite improvements, remaining weaknesses included service access and usage - especially in remote areas, staff retention, workload, and community accountability.

**Conclusions:**

By including perspectives on SRH service provision and BPHS contracting across health-system components and levels, this study contributes to broader debates on the effects of contracting on perceptions and experiences among practitioners and service-users in conflict-affected countries.

## Background

### Health service contracting

Health service provision is particularly challenging in countries affected by or emerging from armed conflict, due to interrelated constraints including destruction of health infrastructure, death and migration of health workers, insufficient domestic resources, and weak governance [1–3]. Non-state providers, particularly international non-governmental organisations (INGOs), often play an important role in addressing health needs during and after conflict [4, 5]. However, these parallel health services are not designed for long-term sustainability, potentially weakening national health systems and consequently undermining the state-building process [6–8].

Linkages between effective service delivery, health system strengthening, and state-building are becoming clearer [2, 3, 7, 9, 10], including the role of non-state providers in this process [4, 5, 11, 12]. However, empirical data remains limited on how the international community can best support the transition from a fragile post-conflict country, often largely dependent on international support, to a sovereign state capable of serving its citizens in an effective and sustainable manner. One approach to improving access to healthcare services after armed conflict, and thereby contributing to the state-building process, is providing a Basic Package of Health Services (BPHS) for all citizens [13–15]. This package prioritises effective primary healthcare interventions (e.g. maternal health, communicable disease control) to address population-level disease burden cost-effectively and equitably [16].

A widespread BPHS delivery mechanism in conflict-affected settings, where government capacity and resources are particularly constrained, is contracting of non-state providers to manage service delivery [17–19]. Providers, contracted through competitive bidding to supply services against predetermined performance targets, are financed, coordinated, and monitored by national governments with support from international donors. BPHS contracting offers potential for rapid scale-up of standardised health services [17, 20, 21]. First introduced nationally in Afghanistan in 2002, BPHS-centred approaches have been adopted in several countries since (e.g. South Sudan, Liberia, Somalia, Sierra Leone, the Democratic Republic of Congo, Timor Leste) [14, 22].

### BPHS in Afghanistan

Community and facility-based primary health services in Afghanistan are provided through the BPHS under stewardship of the Ministry of Public Health (MoPH) [15]. Three major donors fund services in rural areas of all 34 provinces (i.e. USAID in 13 provinces, World Bank in 11 provinces, European Commission in 10 provinces), contracted predominantly through international and national NGOs. BPHS components are (i) maternal and newborn care, (ii) child health and immunisation, (iii) public nutrition, (iv) communicable disease treatment and control, (v) mental health, (vi) disability and physical rehabilitation, and (vii) essential drugs supply.

Afghanistan, the longest-running example of BPHS contracting in a conflict-affected setting, provides a useful case study for exploring how implementation of a national health intervention influences access to prioritised health services [23]. Analysts reported that efforts to improve healthcare access in Afghanistan through the BPHS were constrained by inadequate infrastructure and transportation, restrictive cultural norms, lack of skilled female staff, high out-of-pocket expenditures, reduced access in remote areas and winter months, and inadequate quality of care [24–26].

Routine BPHS evaluation is primarily quantitative, through the Balanced Scorecard approach (e.g. minimum staffing levels achieved, provision of ANC services). Though crucial, this approach remains incomplete. It is limited in its ability to provide explanations for differences in performance or allow for assessment of staff and user perspectives [27]. Inclusion of qualitative research can explore wider systems issues through the perspectives of providers translating policy into practice or service-users experiencing the practical aspects of policy [14, 28]. Very little qualitative research has been conducted to explore whether the BPHS strengthens the health system and builds capacity and leadership within national government [29, 30].

### Study objectives

The study aim was to explore health-system ‘practitioner’ (e.g. policy-makers, managers, frontline providers) and ‘service-user’ perspectives on provision of sexual and reproductive health (SRH) services through BPHS contracting. SRH services were chosen because: (i) SRH cuts across several BPHS components and (ii) SRH was prioritised in both MoPH and donor agendas during BPHS development in Afghanistan due to some of the highest maternal and infant mortality rates in the world having been recorded in the country [27, 31].

The research question, ‘In what ways has the BPHS affected SRH service provision?’ was intentionally broad to capture a diversity of insights. Objectives were to: (i) describe varied health-system perspectives, using the WHO framework and (ii) identify significant perceived improvements and weaknesses in SRH services under the BPHS.

## Methods

### Setting

This study focused on service provision in Bamyan province. Largely rural, with BPHS coverage, it was selected for relative remoteness and research-staff security. Bamyan, in central Afghanistan, is the cultural centre of the Hazara – an ethnic and religious minority that have experienced long-term discrimination. High altitude, rugged terrain, and long winters impede health service provision for most of the 418,500 inhabitants. Three NGOs, IbnSina, Aga Khan Health Services of Afghanistan, and Agency for Assistance and Development of Afghanistan, provide the BPHS with USAID and other funding.

### Study design

A qualitative research design, as described in Lincoln and Guba, incorporated in-depth interviews with health-system practitioners and focus group discussions (FGDs) with service-users [32]. Participants were recruited by AtV and LO. A systems approach, informed by Reid and colleagues’ four-levels model [33], guided participant selection. This model situates healthcare within four interconnected levels: (1) service-users, (2) frontline providers (e.g. doctors, midwives, pharmacists, community health workers), (3) healthcare organisations (e.g. facilities through which healthcare is provided), and (4) healthcare environment (e.g. the political, financial, regulatory regime in which healthcare is organised). Ferlie and Shortell suggest that whether intended changes are bottom-up, top-down, incremental or radical, efforts should address all health-system levels to maximise likelihood for success [34]. For the purposes of this study, SRH services included maternal and newborn health, contraception, and sexually-transmitted infection (STI) and HIV prevention and treatment.

### Data collection

In-depth interviews, using purposive sampling for diversity of opinion, were planned with key informants from three health-system levels who had developed, implemented, or evaluated BPHS services nationally or in Bamyan province: (Reid *et al’s* level 2) frontline providers, (level 3) health facility supervisors and managers, and (level 4) donors and policy-makers – see Table 1. In-depth interviews were conducted in English, face-to-face in Bamyan or Kabul by AtV (in locations selected by participants) or via telephone by LO. Interviews were digitally recorded and transcribed professionally with quality checks by AW and DP.

**Table 1:** **Summary of study participants**

Levels and demographic characteristics	18 interviews (face-to-face)	8 interviews (phone)	4 FGDs (face-to-face)
**Policy-makers/Donors/Advisors (Level 4)**	**7**	**3**	-
Government	3	-	-
UN/Bilateral	3	1	-
CSO/NGO	1	2	-
**Supervisors/Managers (Level 3)**	**7**	**3**	-
Government	3	-	-
UN	1	-	-
CSO/NGO	3	3	-
**Frontline providers (Level 2)**	**4**	**2**	-
Government/NGO	4	2	-
**SRH service-users (Level 1)** ^**a**^			
(a) Bamyan women at a BPHS facility	-	-	1
(b) Bamyan women 2 hrs from a BPHS facility	-	-	1
(c) Non-educated lower-income Kabul women	-	-	1
(d) Educated higher-income Kabul women	-	-	1

**NB:** ^a^BPHS was not implemented in Kabul city. Thus, Kabul women were considered BPHS non-users, Bamyan women attending a BPHS facility were users, and Bamyan women living at a distance from a BPHS facility were considered potential users.

FGDs, using purposive selection for maximum between-group variation, were planned with SRH service-users (i.e. Reid *et al’s* level 1) in rural Bamyan province and urban Kabul city - see Table 1. Kabul service-user FGDs were intended as a pragmatic comparison of reported SRH service access and quality in a populated urban area without BPHS implementation. Bamyan service-user selection was based on distance to nearest BPHS facility, to explore how this might affect service usage in this rural province. Kabul service-user selection was similarly based on distance to nearest referral hospital. Investigators attempted to include a mix of incomes and education levels among FGD participants where possible. FGDs, of approximately 8–12 female participants each, were facilitated in Dari by NA. FGDs were digitally recorded and transcribed and translated by AS.

### Data analysis

Inductive thematic coding used the WHO Health System Framework indicators (i.e. service delivery, workforce, health information, access to medicines, financing, stewardship), as the BPHS is a horizontal approach that affects the whole health system and many practitioners are familiar with the WHO framework [35]. Two authors separately applied a preliminary coding framework to the first transcript. Coding was compared for consistency and the framework adapted as necessary. Once the final coding framework was agreed, remaining interviews were divided and coded. Reporting adhered to RATS criteria for qualitative research [36].

### Ethics

All participants received and were read study information sheets and written or verbal informed consent was recorded. Interviews and FGDs were conducted confidentially and recorded anonymously using numerical identification. Ethics approval was provided by the MoPH Institutional Review Board in Afghanistan and the London School of Hygiene & Tropical Medicine Research Ethics Committee in the United Kingdom.

## Results

Twenty-six in-depth interviews were conducted with health-system practitioners (i.e. 18 face-to-face, eight phone) in 2010 and 2012. Nine potential interviewees did not respond to invitation or follow-up, but did not obviously differ from interviewees with respect to job description or gender. Interviews in 2012 explored initial access and accountability findings in more depth. Six participants were primarily frontline providers (i.e. Reid *et al*’s level 2). Ten participants were primarily supervisors and managers (i.e. level 3), though four reported additional frontline duties and seven reported some policy-influencing activities. Ten were primarily policy-makers and advisors (i.e. level 4), though five reported additional managerial responsibilities. Demographically, 72% were Afghan, 49% female, and they worked as UN/bilateral donors or technical advisors (19%), government staff (46%), and/or civil society/non-governmental organisation (CSO/NGO) staff (58%). There was considerable overlap, particularly between government and CSO/NGO staff – see Table 1.

**Figure 1:**
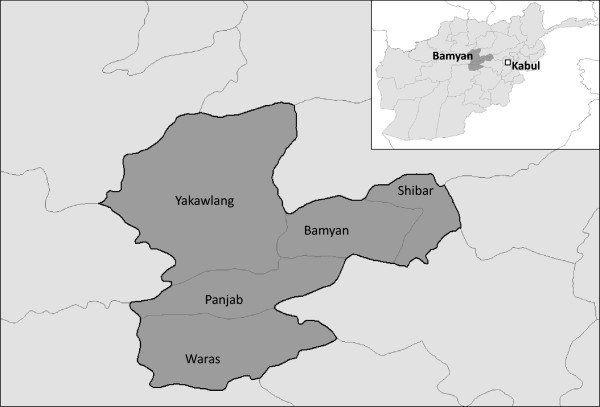
**Bamyan Districts map.** Source: Katharine Footman

Four FGDs with SRH service-users were conducted in 2009–2010. Two in Bamyan province included: (a) higher-income women presenting at a Basic Health Clinic in Bamyan district (i.e. ‘BPHS users’), and (b) lower-income women in a village two hours walk from the nearest BPHS facility in Shibar district (i.e. ‘potential BPHS users’) - see Figure 1. Two in Kabul city included: (c) non-educated low-income female SRH service-users living in District 1 Kabul, and (d) somewhat-educated higher-income female SRH service-users living in District 4 Kabul. All women recruited agreed to participate. Additional planned FGDs were not conducted due to funding constraints.

Results are reported under five WHO health system categories, sub-categorised by emergent themes. ‘*Access to medicines’* was excluded as no participants discussed this in relation to SRH. Perspectives of health-system practitioners (i.e. Reid *et al*’s levels 2–4) and SRH service-users (i.e. level 1) are reported separately, as authors found a natural dichotomy between these perspectives. Unless otherwise indicated, service-user quotes are from Bamyan FGDs.

### Service delivery

#### Coverage and access

Health-system practitioners reported increased SRH coverage under BPHS contracting.

*“With the introduction of the BPHS, that was simultaneous with the attention of the international community to Afghanistan…There were areas, districts, where we did not have even a single vaccinator, but now they have 45 health centres in those districts”.* (Donor-level 4)

Despite improved coverage and better security, facility access in parts of Bamyan remained challenging. Female health-workers and mothers living in hard-to-reach areas were particularly hampered by distance, poor roads, and lack of transportation. One midwife commented this reduced her ability to respond to pregnancy complications.

*“Half of the work of the clinic involves deliveries. When you ask people ‘why don’t you go to the clinic?’ they say it’s too far”.* (BPHS provider-level 2)

Ambulances and mobile outreach helped address distance. Ambulances were available at larger BPHS facilities, although limited mobile phone coverage reduced their effectiveness. Villages at least three-hours walk from the nearest health facility received visits from Integrated Maternal and Child Health and Nutrition (IMCHN) units. However, access worsened significantly during Bamyan’s six-month winter:

*“But during the winter, the roads are blocked…Nothing can go there except helicopters. Even helicopters cannot go there sometimes”.* (BPHS manager-level 3)

Initiatives to provide healthcare for winter-bound communities included community midwifery training:

*“In winter-time for IMCHN we even go by horse in some cases. We change our plan if we cannot go to some of those blocked villages…The idea now is to train some midwives for every 1,000-1,500 people…They will stay there”.* (BPHS provider-level 2)

### Demand and usage

Bamyan providers reported increased demand and usage of SRH services under BPHS contracting - particularly demand for family planning services, which reportedly “increased by two-fold” even among those living at greater distances from facilities.

*“They are willing to come to the health facilities, almost all of them are not sick. Most of them need the services, the reproductive health services…They come because they are aware these are useful for them”.* (BPHS provider-level 2)

Despite increased demand, family-planning misconceptions remained:

*“Let’s say someone has bleeding. She somehow thinks it’s related to her taking birth-control. That scares many more women to sign up for family planning…Some people even think that using birth control will prevent them from having sex or that it reduces their drive”.* (BPHS manager-level 3)

Practitioners reported minimal increases in STI services usage despite needs. Providers described patients as ‘shy’ about discussing sensitive health issues, potentially contributing to STI treatment-seeking delays.

“*Most of the patients that come here have some form of STIs. NGOs and the aid community have not really paid any attention to this particular issue…”* (BPHS provider-level 2)

Practitioners similarly explained low numbers of facility deliveries as cultural preference, advocating better health promotion and ‘trust-building’ efforts to increase service demand and usage.

“*Even when there is a skilled birth attendant in the health facility they prefer to stay home”.* (BPHS manager-level 3)

Perceived socio-cultural barriers to SRH service usage included female role expectations.

*“Women should be working for their family members like going to the land to harvest, taking important daily activities of their family, and even bringing income to the family. Condemned to stay at home and still not go to school. And if they graduate from school, not to university”.* (BPHS provider-level 2)

#### Service-user perspectives

Service-users in Bamyan reported improved SRH service coverage and access since establishment of the local BPHS clinic. Previously, they either attended a hospital that was “very far” by ambulance or car or were treated and delivered at home.

*“If the BPHS clinic was not there we would have to walk for hours to get to another health facility or hospital”* (BPHS user-level 1)

Distance-related differences in service usage were reported, with nearby women using services routinely and distant women using them for emergencies only. Distant participants usually delivered at home with a *daya* (i.e. traditional birth attendant)*.*

*“People are poor and they have to stay at home…”* (Potential BPHS user-level 1)

When asked if they preferred to give birth at home or clinic, women unanimously responded the latter if feasible.

“*The daya is not as knowledgeable as the clinic”.* (Potential BPHS user-level 1)

Distance-related knowledge differences were also apparent. FGD participants living near a BPHS facility – and thus more likely to use services – could identify several family planning methods and expressed positive attitudes towards modern contraceptives, birth spacing, and not having ‘too many’ children. FGD participants living two-hours from a BPHS facility reported that their family planning knowledge was poor, contraceptives could lead to infertility, and pregnancies could not be planned but ‘just happen’.

Women in both Bamyan and Kabul identified social demands, particularly childcare, as barriers to SRH service usage.

*“My child is three months old. I was very ill during my pregnancy…, but I was not able to go to the clinic because I do not have anybody to watch my children”.* (Higher-income service-user, Kabul-level 1)

### Workforce

#### Gender and numbers

Health-system practitioners reported that improvements in coverage and usage reflected increased provider numbers and quality, particularly female staff numbers.

*“I think one of the best things that the partners and the MoPH have done is promoting services of women by a woman”.* (Donor-Level 4*)*

However, more female staff - across all SRH cadres (e.g. doctors, nurses, midwives, CHWs) - were reportedly required. For example, at least two midwives were required to provide 24-hour basic emergency obstetric care.

“*At the BPHS level in a BHC [Basic Health Centre] we find the midwives 24-hours on duty. These midwives need rest, but she cannot leave at all because she’s the only one at the health facility…”* (Civil society leader-level 4)

#### Training and standards

Practitioners agreed a need for continued capacity-building and training of government staff at all levels. Perceptions of improved service quality were often framed in terms of increased training and standardisation.

“*Right now there are specific standards covering antenatal care, prenatal care, postnatal care, safe delivery or complicated cases. This is only good for staff, right now they know what to do and how to apply it”.* (Donor-level 4)

Midwifery training was particularly praised.

*“In 2002 in our country we had 467 midwives, but now we have more than 2,600…”* (MoPH trainer-level 3)

#### Role and workload

Community-based care, an important BPHS component, is most visible as female community health workers (CHWs). Practitioners generally reflected positively on this, with CHWs playing an important role in contraceptives provision and community trust-building (e.g. encouraging facility deliveries). However, several acknowledged CHW capacity was low and expected workload significant.

*“I think there are a lot of problems with the CHW. It has its benefits of course. In a situation where you have nothing, this is a very good network, but most of the CHWs, if you see their education background, they are very low and they don’t have this much ability to recognise the need of the patient or to give the medicine”.* (MoPH senior manager-level 3)

Despite low CHW capacity and heavy workload, some argued that CHW roles could be expanded to include full antenatal care (e.g. blood pressure, weight measurement) and even uncomplicated deliveries. Others acknowledged the challenges of motivating volunteers.

*“Each health post covers 100–150 families…[and] two CHWs. They are volunteers. Not all of them are able to leave their life and work for these numbers of families. So it was not as much effective as it was thought”.* (BPHS manager-level 3)

#### Retention

Increased training opportunities combined with an improved job market resulted in attrition of qualified staff, particularly female staff in rural areas:

*“But now the market is better, quite better than before…You see as soon as a person is qualified, he is not willing to work in that position anymore. And one of the major problems in the health facilities, the competent staff, specially the high level staff like female MD is quite scarce”.* (BPHS manager-level 3)

High staff turnover was reportedly problematic.

*“…there are also people that move from one province to the other because they want to get to a province where, for example, the family is better off, there are better schools, etc.”* (Donor-level 4*)*

Service-user perspectives

Service-users reiterated practitioner views that more health workers were needed, particularly clinical staff (e.g. doctors, nurses), though they did not specify female providers.

### Health information

#### Monitoring and evaluation

Practitioners reported improved SRH monitoring and evaluation under the BPHS, most acknowledging the combined effort of donors, MoPH, and NGO sub-contractors.

*“Because from 9 indicators, most of them are about reproductive health”.* (BPHS provider-level 2)

Several described implementation of the balanced scorecard, annual national monitoring checklist, and increased research and documentation as evidence of improvement. Project managers and donors focussed on the need for additional indicators (e.g. community-based activities, supervision of new health workers), while providers focussed on the data collection burden of existing indicators and reporting mechanisms.

*“So these different types of tools are coming and going. This is a little boring, so we need to have a fixed tool because time is wasting…”* (MoPH manager-level 3*)*

#### Quality measurement

One manager said the balanced-scorecard could not detect ‘real’ service quality. However, most practitioners discussed the difficulties of determining whether quality of care had improved, due to reporting inconsistencies and perceived data inaccuracies.

*“It is six months or more that nobody has come from MoPH…Sometimes, you see, people do not provide the real information and the real feedback because he has connection with somebody”.* (BPHS provider-level 2)

While one donor said improving SRH services quality measurement was “an immediate need,” most practitioners said quality improvements “just take time”.

### Financing

#### Financial information

Practitioners agreed funding for SRH services had increased under the BPHS:

*“From the level of funding that we had in 2003, now it's increased tremendously…”* (MoPH senior manager-level 4*)*

Several said more SRH-specific funding was needed to further improve and expand SRH service delivery, while others favoured funding all primary healthcare needs equally. Tracking of BPHS component funding remained challenging.

*“We don’t have a health financing information system…[and] don’t know exactly which percentage will move to the specific lines in the reproductive health”.* (NGO manager-level 3*)*

Some donors admitted financial tracking challenges:

*“Of course, if we [donors] all had the same budget headings, it will be much easier, so I think it is possible but it has not been looked at”.* (Donor-level 4*)*

#### Incentives

Practitioners suggested flexible financial incentives could help retain health-workers in challenging areas. Bamyan practitioners suggested an incentive system could encourage female doctors from rural areas to return after medical school, though national standardisation of salary policy minimised opportunities for such concessions. An NGO advisor noted that rural health-workers were generally less satisfied about their salaries.

*“If you ask a women to go and provide services to a rural area, they may hesitate to go. But if they have an internal mechanism that they go for three or four years and they receive high compensation they go and the condition will be that they train local women in that area in three years, so after three years they have another person in place”.* (NGO manager-level 3)

Bamyan practitioners suggested financial incentives could improve CHW motivation. CHWs, though officially volunteers, were sometimes reimbursed for delivery referrals using non-BPHS funding. Both CHWs and *dayas* often received informal payments from families.

Non-financial incentives were suggested to encourage service-user attendance (e.g. antenatal care).

*“Incentives like material and blankets for the newborn baby, like a kit for a woman that comes to postnatal care for the second and third visits. Now almost all of them do not come for the second or third visits”.* (BPHS provider-level 2)

#### Service-user perspectives

SRH users reported travel expenses, and fees in non-BPHS areas, limited access. Bamyan service-users confirmed they were not charged for health services, but travel costs could be significant.

*“So if there are problems, they bring a car from Bamyan or closer districts. They walk on the main road and wave a car to help us out. It costs 2,000-5,000 AFS [2013 US$40-100] for a car to take us to the clinic”.* (Potential BPHS user-Level 1)

Kabul service-users identified financial barriers as most significant in receiving care. Out-of-pocket expenditures were reported as particularly difficult in this non-BPHS area, even for higher-income users and despite regulations stipulating services in public facilities be provided free-of-charge.

*“Even if I go to the public hospital or clinic, I cannot afford to buy the medicine. Money is needed for doctors’ fees, medicine, and travel”.* (Higher-income service-user, Kabul-level 1)

### Stewardship

#### Prioritisation

Health-system practitioners agreed that MoPH took the lead in BPHS development, with significant support from international donors. Practitioners identified SRH as a donor priority during BPHS design:

“*If the RH component was not there, I'm not sure we would have got the commitment of donors. So…BPHS got funding because of RH. I can say RH got funding because of BPHS”. (*MoPH senior manager-level 4*)*

Reasons given for donor commitment to the BPHS included donor involvement in development (i.e. felt ownership), outcome-oriented design and measurement, and accepted effectiveness of selected interventions (e.g. emergency obstetric care). While expert stakeholder advice guided SRH prioritisation, most identified a 2002 maternal mortality study - showing Afghanistan had the second highest rate worldwide [37, 38] - as key:

*“Because only talking about the problem is not enough, you have to provide evidence”. (*MoPH senior manager-level 4*)*

Most expected SRH to remain a BPHS priority even if new components were introduced (e.g. mental health, disability). SRH components were considered equally important, though some identified maternal care as a focus, followed by family planning. A government official noted strong advocacy for community midwifery education (CME) within MoPH, while several noted that gender violence and equity remained sensitive issues.

#### Coordination

Practitioners agreed that health-system coordination had improved during BPHS implementation. Cooperation between MoPH and other stakeholders (e.g. NGOs, UN agencies, donors) was noted during national and provincial planning and BPHS revisions. However, coordination challenges were identified for health information, primary and secondary healthcare, provincial and district hospitals, public and private sectors, and donors.

Practitioners noted that health information management at government and facility levels could be more effectively coordinated (e.g. data sharing between the SRH directorate and MoPH, cooperation between Maternal Health Units and Integrated Maternal/Child Health Nutrition units). Greater coordination between MoPH and other ministries (e.g. Ministry of Economic Planning, Ministry of Education) would improve accountability.

*“I think both [MoPH and Ministry of Education] should work together. [For example] if teenagers receive sex education [SRH curriculum] only in schools but not in the clinics, then there will still be a gap”.* (BPHS manager-level 3)

Primary and secondary healthcare coordination required further strengthening, with the referral system - particularly for obstetric complications – still regarded as weak.

*“So there’s the BPHS and the EPHS and they should complement one another in that there should be a functional referral system but there’s still a lot of work to be done on that…I’ve seen women carried for four days by men from the village to come to the hospital for obstructed labour”.* (NGO provider-level 2)

One practitioner noted the lack of night-time communication between district and provincial hospitals (i.e. radio operators worked during the day). Another explained that patients with complications went to the provincial hospital, avoiding health facilities.

*“The referral system still is weak, referral from the health facilities to the district hospital, specifically for major obstetric complications”.* (NGO manager-level 3)

Several emphasised donor coordination:

“*Coordination is always difficult, especially now with the different donors funding it, every donor having its own procedures and its own reporting requirements”.* (MoPH senior manager-level 4*)*

#### Accountability

Most practitioners said the BPHS, particularly contracting-out mechanisms, had improved accountability to MoPH and donors.

*“I think the implementers are more accountable because they are contracted…”* (Donor-level 4*)*

However, several expressed uncertainty about the extent of accountability to communities. One senior manager said limited education reduced community self-advocacy, while others reported the BPHS stimulated community involvement.

*“It’s a good mechanism for accountability towards the donors, towards the MoPH, towards the institutions, how much this is accountable to the beneficiaries, I don’t know”.* (Donor-level 4*)*

#### Service-user perspectives

Many service-users noted progress. One BPHS-user mentioned that mobile phones and community ambulances enabled families to arrange their own hospital transport. However, perspectives also reflected discrepancies between donor and community accountability. Women reported not knowing whom to contact should they experience problems with BPHS staff, making accountability at this level challenging.

*“I am uneducated and I can’t read and write. First we go to God. But if we know someone can help us, then we would go to him. Maybe the head of Shura or whoever…” (*BPHS user-level 1)

## Discussion

### Improvements and weaknesses

Improvements in Bamyan were noted by participants in all five health-system components discussed, with key perceived improvements identified in SRH service delivery, particularly coverage and SRH workforce (e.g. in improved gender balance, numbers, training, standardisation). Important remaining weaknesses included service access, usage, staff retention and workload. Community accountability findings were equivocal.

#### Service delivery

Many service-users and potential users still considered access challenging. Despite increased facilities, travel restrictions due to security, cost, and distance (e.g. poor roads, expensive/unavailable transport, dangerous weather) still influenced care-seeking choices in Bamyan. A travel reimbursement system, such as that piloted in Badakhshan province, and improved ambulance services could reduce travel cost and availability barriers, particularly for obstetric emergencies. Strengthened community-based services (e.g. community midwives, CHWs) could help reduce security and weather-related travel barriers.

There were notable disparities between practitioner and service-user responses on facility deliveries, with practitioners emphasising cultural barriers and service-users discussing logistical difficulties with access. Unfortunately, the reduced number of FGDs did not allow for deeper exploration. However, Speakman and colleagues report increased usage of skilled birth attendants once they became available in communities, suggesting that demand for safer deliveries may outweigh perceived cultural barriers in rural areas [38]. Follow-up is needed to determine the most effective approaches to promoting facility deliveries and skilled attendance at every birth.

#### Workforce

Retention is challenging when staff have attractive opportunities elsewhere. It is perhaps unsurprising that rural and remote postings were unpopular with many providers, yet as Petit and others note, the BPHS depends on rural health-staff for community-based services [14, 16, 24]. Lack of appropriate incentivisation, recruitment and retention – particularly of female staff – will likely challenge scale-up of BPHS services at both community and facility levels [39]. Workload, particularly for midwives and CHWs, is a related challenge. CHW acceptability remains an issue, with some policy-makers advocating increasing their role beyond family planning advice and referral and others questioning their capacity. CHWs are not a solution for life-threatening cases nor could they replace skilled birth attendants. However, their remit is likely to expand given the continued need for low-cost primary healthcare [39].

#### Health information and financing

Health information and financing for SRH services, despite ongoing challenges, appear to have improved since BPHS initiation, due to significant international financial and technical support [23, 40, 41]. Among SRH service-users, out-of-pocket expenditures in non-BPHS areas were an important access barrier, suggesting the initial BPHS focus on under-served rural areas did increase access in Bamyan [24–26, 42]. Authors support the 2012 BPHS expansion to urban areas, including Kabul city, as a way to increase health equity.

#### Stewardship

Weak community accountability is not uncommon in fragile contexts such as Afghanistan, as governance structures are often insufficient for the ‘long route’ of accountability to be effective (i.e. citizens holding the state accountable using their ‘voice’ , via elections or advocacy activities, and the state ensuring providers fulfil agreed responsibilities) [43–47]. However, findings on community accountability were equivocal. Accounts indicated uncertainty among service-users about accountability mechanisms, without clarifying whether mechanisms existed or not. Follow-up is needed to provide additional insight.

### Limitations

Due to time and security constraints, a reduced number of interviews and FGDs were conducted. This limited numbers of health-system perspectives, particularly those of CHWs and service-users, and restricted exploration of differences between Bamyan service-users. Interviews and FGDs focussed on health services in Bamyan, a culturally and geographically unique province, thus results are not generalisable across Afghanistan. The reality that changes take time was demonstrated in 2012 follow-up interviews, in which the same problems were discussed and results found as in 2009–2010 interviews.

## Conclusions

BPHS contracting is increasing in post-conflict settings, with some notable success in improving SRH access and outcomes [14, 48]. This study contributes perspectives on SRH service implementation through BPHS contracting across health-system components and levels in Afghanistan. Some findings were to be expected (e.g. continued geographical access difficulties, increased health workforce and retention challenges, improved health information), while others were perhaps more surprising (e.g. practitioner and service-user differences in reported delivery preferences, the significance of financial barriers in non-BPHS areas - even among relatively higher-income service users). This exploration of SRH services delivery in Bamyan contributes to broader debates on the effects of health service contracting on perceptions and experiences of health services provision among practitioners and service-users in conflict-affected areas [7, 14].
